# Bacterium-Mediated RNA Interference: Potential Application in Plant Protection

**DOI:** 10.3390/plants8120572

**Published:** 2019-12-05

**Authors:** Simon Goodfellow, Daai Zhang, Ming-Bo Wang, Ren Zhang

**Affiliations:** 1 School of Chemistry and Molecular Bioscience, University of Wollongong, NSW 2522, Australia; 2 CSIRO Agriculture and Food, Canberra, ACT 2601, Australia

**Keywords:** RNA interference, plant protection, disease control, bacterial-mediated RNAi

## Abstract

RNAi has emerged as a promising tool for targeting agricultural pests and pathogens and could provide an environmentally friendly alternative to traditional means of control. However, the deployment of this technology is still limited by a lack of suitable exogenous- or externally applied delivery mechanisms. Numerous means of overcoming this limitation are being explored. One such method, bacterium-mediated RNA interference, or bmRNAi, has been explored in other systems and shows great potential for application to agriculture. Here, we review the current state of bmRNAi, examine the technical limitations and possible improvements, and discuss its potential applications in crop protection.

## 1. Introduction

RNA interference (RNAi) refers to the down-regulation of gene expression through double-stranded (dsRNA), or hairpin RNA (hpRNA)-induced RNA degradation in eukaryotes. Since its discovery in 1998, RNAi has rapidly proven to be a valuable tool in genetic engineering [1,2], as well as an essential mechanism of molecular defense [3] and gene regulation [4,5].

RNAi is also coming to prominence as a versatile and environmentally friendly alternative to traditional means of agricultural pest and disease control, as dsRNA expressed by or applied to plants can silence microbial eukaryote or invertebrate genes with high specificity. This potential is however somewhat limited by a lack of a rapid, flexible, and effective means of introducing precursor dsRNA. Such techniques fall broadly into two categories: endogenous expression—where a gene encoding a dsRNA is stably or transiently expressed in the target organism, and exogenous application—where effector RNAi molecules are produced outside of and are taken up into the target organisms.

Endogenous, or transformation application, while reliable and thereby the most commonly used method, is not without its limitations. Transgene introductions are time consuming and testing of new dsRNA in plant lines expressing them can require considerable expertise and resources. Plant transformation is also limited in terms of host-range and is untested or presently impossible in many species [6]. This is further compounded by the capacity of many plants to self-silence dsRNA-encoding genes through RNA-directed DNA methylation, resulting in unstable or transient silencing of the target gene [7]. 

Exogenous application overcomes some of the constraints associated with transforming plants by having dsRNA synthesized outside of the target. dsRNA produced from both biological [8] and chemical sources [9] can be applied to plants through foliar application, such as spraying [10], or infiltration [11]. In this manner, both biologically and chemically produced dsRNA can induce the silencing of plant, viral [12], fungal [13], and invertebrate genes [14]. When possible, this greatly speeds up the process of testing new dsRNA by allowing for targets to be tested simultaneously without having to generate individual plant lines. This method is, however, potentially limited by the transient nature of the effect, in that sustained silencing or protection may require repeated applications [15].

In this communication, we provide a brief overview on the current state of exogenous RNAi against pests and pathogens in host organisms, especially plants. We will then focus on bacterium- mediated RNAi (bmRNAi), a novel exogenous delivery method that uses live hpRNA-expressing bacteria that promises to overcome some of the issues encountered by previous methods. This will involve design considerations, as well as precedents in mammalian and insect models, followed by examining the potential for a test system in plants. Finally, we will briefly discuss potential applications and the utility of bmRNAi as a selective and environmentally friendly biocide against plant diseases and pests.

## 2. Exo-RNAi in Plant Protection

Exogenous RNAi (Exo-RNAi), or the application of externally synthesized RNAi effectors has been shown to be a promising alternative method of RNAi delivery to genetic transformation approaches in a wide variety of organisms. dsRNA applied in this manner enters plant tissues and produces a temporary downregulation of targeted genes. Exo-RNAi also has potential as a novel crop protectant, as exogenously-administered dsRNA can also silence genes of virus, fungi, and insects in association with the plant [15].

Synthesizing RNAi effectors outside of eukaryotic hosts comes with a range of benefits. As said, endogenous RNAi requires the often lengthy and difficult process of transforming and screening plant lines that optimally and stably express active RNA [16], which is further compounded by RNA-induced self-silencing of dsRNA transgenes [17]. Furthermore, RNAi-induced insect pest resistance requires the accumulation of long dsRNA, which occurs at low levels in transgenic plants, due to processing by plant RNAi machineries [18]. These difficulties can be bypassed in Exo-RNAi by using either chemical [19], in-vitro [20], or biological syntheses of dsRNA with a desired length [21,22], which are simpler to customize and prepare. Both in-vitro and chemical synthesis are widely commercially available. Such technologies, while more expensive than biological methods, allow for the rapid and reliable production of large quantities of very pure RNAi molecules. Additionally, these permit the introduction of numerous chemical modifications [22,23] and while these are not readily producible for most labs, can be rapidly and easily outsourced. 

Biological synthesis methods typically utilize double-stranded RNase-deficient bacterial strains, which are transformed with hpRNA or dsRNA-expressing genes [21]. These strains are cultured in growth media, followed by the harvesting of cells, and optionally, the extraction of the resultant RNA. RNA produced by these means are effective without extensive purification and can be directly applied to plants as heat-killed bacteria [24] or lysate [8]. This method, while slower to customize due to the labor associated with producing and cloning new RNA-generating genes into bacteria, is far cheaper, especially for long term or high-volume applications, where a large amount of silencing RNA is required.

Exogenously-produced dsRNA has been applied to plants and plant-associated organisms in numerous ways. Both surface treatments, such as spraying [25] and soaking [26], as well as invasive methods, such as infiltration [11] and injection [27,28], have been used in previous studies. Surface treatments typically deposit the RNA onto the plant, where it coats treated tissues and is taken up by the host plant, and by organisms associated with [29], or feeding on them [26]. In contrast, invasive treatments introduce RNA indirectly into systemic circulation, relying instead on the vascular and local movement of RNA into pest-challenged tissue [27,28].

The fate and movement of Exo-RNAi molecules inside plants is still an area of active study, although it has been shown that once absorbed, plants process dsRNA, such as hpRNA into siRNA, which silence genes locally or are mobilized to distal cells via both plasmodesmata and the vascular system. Beyond this it has also been shown that these can be used as templates or inducer to produce further dsRNA and hence siRNA via RNA-dependent RNA polymerases (RdRPs), allowing the silencing signal to propagate and increase in strength [30,31]. 

The action of RNAi biocides varies with the chosen target and setting, due to differences in the ways that organisms interact with plant-associated RNA. There is presently little known in terms of where it is encountered, how it is internalized and how it generates a protective effect from gene silencing. On a broad level, this includes the differences between molecular parasites, such as viruses, which require an intracellular and plant-mediated response [32], and eukaryotic parasites, like fungi and insects, which instead need to ingest and integrate dsRNA into their own gene silencing proteins to enact an effect [33,34]. Individual species of pests and pathogens also vary greatly in this respect, due to differences in the feeding ecology, physiology, pathology, and RNA metabolism, such that, even within one taxon, such as insects or fungi, multiple different silencing and delivery strategies are required, and effective RNAi is usually limited to a small number of species [14,33,35,36,37]. 

Likewise, gene targets must also be considered in terms of both the specificity and effect desired. RNAi constructs, foremost, need to target a gene essential enough to the pest or pathogen, such that it can inhibit its growth, reproduction, or survival. This exercise has been largely successful—previous studies have been able to produce a wide range of effects with potential for use in RNAi crop protection, some notable examples of these are presented in Table 1. The portion of the gene chosen also determines specificity—broad spectrum applications require conserved regions present within multiple related species [38], while more specific applications can instead utilize variable stretches that differ on the species or even population level [29]. In this manner, it may be possible to tune the specificity of an Exo-RNAi agent to the desired taxonomic level, and with careful consideration of both the host, target, and context, allow the application without off-target effects.

One unifying feature of exogenous RNAi strategies are the difficulties associated with degradation and uptake of dsRNA. Whether inside the plant or deposited on it, RNAi molecules can be broken down by physical and biological means and consequently lose their ability to protect the plant. Beyond this, the uptake of silencing RNA into targeted organisms, either by ingestion, or absorption also limits the effective RNA concentration. This is especially relevant in cases where the dsRNA needs to be taken up from the digestive tract, as with insects, which commonly possess gut-specific ribonucleases capable of degrading effectors. These issues have been addressed to some degree through the development of compounds and materials that bind RNA molecules to increase their stability, adhesion to plant tissues, and subsequent uptake. Notable innovations in this area include the use of layered double hydroxide clay nanosheets [9], carrier peptides [11], surfactants [25] and cationic nanoparticles [39]. However, it is worth noting that widespread application of these agents has been lacking in plants and are often confined to single pests or pathogen model systems.

### 2.1. Virus

RNA-induced viral defense induction is highly conserved in plants and, in addition to its stated role, also forms a critical component of immune responses against other molecular parasites such as transposons. In a natural context, dsRNA and hpRNA produced as intermediates of viral transcription and replication are processed into 21–24 nt small interfering RNA (siRNA) by the RNAi pathways present in the plant host and subsequently used as guide for the degradation or methylation of viral sequences [49]. This functions systemically, and siRNA produced in response to local infection is transported both between adjacent cells and the vascular system to protect distal tissue from further infection [50]. The artificial counterpart functions in a similar manner; artificial RNAi molecules introduced by either transformation, or exogenous means to target viral genes have been shown to be able to reduce viral accumulation and the severity of associated symptoms [51].

Antiviral exoRNAi studies have, to date, only applied RNAi molecules either by spray [9,12,52] or abrasive-assisted mechanical inoculation [45,53,54,55,56,57,58,59], and while it is highly likely that other delivery mechanisms have this capacity, they remain to be tested. Numerous genes are appropriate as antiviral targets in both host-induced gene silencing (HIGS) and exoRNAi systems, with those encoding viral replicases [8], coat proteins [56], or viral RNAi suppressors [45] being the most common. Demonstrations of this method have successfully used numerous species of RNAi molecules including dsRNA, hpRNA, siRNA, and ssRNA—although longer and more complex species are more effective [59]. The protection typically generated by a single treatment has been shown to last between 2 and 7 days, although with the addition of protective agents—such as clay nanosheets—this can be extended up to 70 days [52].

### 2.2. Fungi

RNAi has also been shown to form a component of fungal pathogenesis and its cognate defense by the plant. In line with this, several studies have now shown that RNA is reciprocally transmissible between fungi and plants in an offence and defense role respectively—and that these can have an influence on the progression and severity of fungal infection [60]. The defense aspect of this interaction has been extensively explored in recent years and led to the development of numerous antifungal HIGS systems capable of artificially replicating this effect [33]. These are however, limited by the need to transform plants but nonetheless demonstrate the potential for a broad range of targets and applicability of RNAi to fungal pathogen management.

Exogenous RNAi technologies—such as dsRNA sprays, or bacterial mediation also have promise as a potential non-genetic transformation-based means to leverage antifungal HIGS findings—several studies have shown that a similar level of protection, albeit temporary, can be produced using foliar-applied dsRNA and siRNA. RNA applied in this way are believed to produce antifungal resistance by two mechanisms—the first involves direct interaction between fungi and dsRNA present in their environment, which are absorbed and subsequently silence the targeted gene [46]. The second pathway is believed to involve RNA-dependent RNA polymerases—some of the dsRNA applied is taken up by the plant and subsequently used as a template or inducer for systemic secondary siRNA production for several days following an initial treatment. This secondary siRNA retains some of the protective activity of the original dsRNAs and can be transported into fungal cells—potentially extending the window of protection relative to surface-deposited RNA [10,61].

There are limited precedents for antifungal exoRNAi and, as such, many application methods and gene targets remain to be tested. The research that does exist has shown that spraying [62] or dropping [46] dsRNA or siRNA onto leaf surfaces are viable methods of inoculation. Additionally, silencing genes associated with cell wall morphogenesis [61], fungicide resistance [63], or pathogenesis, including proteins involved in suppressing host immunity [46], can reduce the severity and spread of infection. This effect is maintained between 2 and 7 dpi (days post inoculation), however this window can be extended up to 14 dpi when co-administered with a fungicide [62,63].

### 2.3. Insects

RNAi has emerged in recent years as a potential alternative to conventional pesticides—many studies have now demonstrated that dsRNA fed or applied to insects can induce the systemic downregulation of targeted genes. Moreover, it has been shown that meaningful reductions in survival and fecundity can be produced by targeting essential genes in this manner [64]. Numerous application methods have been tested for insecticidal RNA in plants, including foliar/spray application—both to the plant [26] and to insects on the plant [65,66], as well as injection and root soaking [67,68]. As with fungi, once applied, the RNAi molecules (usually long dsRNA) present can induce insecticidal silencing, either by direct contact with the desired species, or by interaction with systemically spread siRNA—using the plant as an intermediate [69]. An interesting difference between these two cases is the lack of a well-documented secondary siRNA system—homologues of plant and fungal RdRPs have not been found in insects or humans [70]. This however, does not preclude the possibility of RNAi being amplified in insects—the prolonged and systemic effect mediated by single dsRNA treatments in insects is suggestive of another route of systemic amplification [64].

In a general sense, once the insect ingests the dsRNA payload, a portion of it passes from the gut into systemic circulation in the haemolymph—from which cells absorb dsRNA and enact silencing [37]. The absorption of dietary dsRNA in insects is believed to occur via two mechanisms—transmembrane channels or receptor-mediated endocytosis. The former pathway involves two proteins—SID-1 (Systemic RNAi defective—1) and SID-2. These internalize dsRNA in two steps, SID2 facilitates the uptake of dsRNA from the intestinal lumen, at which point SID1 catalyzes transport into the cytosol [71,72,73]. The endocytic mechanism instead functions by scavenger-receptors wherein dsRNA is bound by transmembrane receptors and internalized into an endosome. Previous research to elucidate this mechanism in *Drosophila melanogaster* identified two such proteins—SR-CI (Scavenger Receptor—CI) and Eater—which were found to be responsible for up to 90% of cellular RNA uptake [74]. Additionally—it has also been shown that dsRNA can be absorbed through the cuticle at a high enough rate to mediate an effect—suggesting that foliar treatments may also have promise [65].

The window of protection offered by insecticidal exoRNAi is variable—some experiments have documented effects on insects at 28 dpt (days post treatment) [26], while others have only demonstrated protection between 3 and 7 dpt [75]. Additionally, the systemic spread of protective RNAi has not been demonstrated in all studies, while a study in tomato demonstrated systemic spread of dsRNA [76], a similar one performed in potato did not [26]. Another major complication in the application of exoRNAi to insects is the presence of extracellular RNases in both the gut and hemolymph of insects which have been shown to attenuate the silencing effect in some species by prematurely degrading RNAi effectors [24]. This has been addressed in some previous studies, by silencing RNases simultaneously, although nonetheless represents a barrier to broad application [77].

While these precedents all show the potential efficacy of exogenously-produced dsRNA as crop protectants, there are, nonetheless, many challenges that need to be addressed before it can be considered an alternative to genetic transformation methods in agricultural settings. Both dsRNA degradation and entry to the target cell remain as significant barriers in this respect. In all cases regular repeated applications are required to maintain dsRNA levels high enough to produce an effect, which is both laborious and costly. As stated previously, significant improvements have been made with the addition of agents to improve both traits, although these have yet to be implemented beyond the laboratory. Using live bacteria, or bacterium-mediated RNAi is a promising alternative to these approaches and could allow for the delivery of more consistent and higher doses of RNA, for longer periods of time than possible without in situ synthesis. In addition, this method may also be able to improve dsRNA uptake and longevity by facilitating its entrance to the cell [43], and perhaps insulating dsRNA from extracellular RNases, respectively.

## 3. BmRNAi—Bacterium-Mediated RNA Interference

Bacterium-mediated RNA interference (bmRNAi) refers to a delivery method wherein live bacteria expressing dsRNA are applied to and colonize an organism to produce and facilitate the uptake of dsRNA in situ, resulting in RNAi knockdown of the targeted gene [78,79]. This method has been shown to be effective in both mammalian cell culture and animal subjects and shows the potential to overcome the weaknesses of more-traditional endogenous and exogenous application strategies by increasing the duration and strength of the silencing effect, while reducing both the time and effort required to produce knockdowns. There has been no bmRNAi research reported in plants.

Using bacteria to continuously apply RNAi has several advantages, not least of which is the reduced difficulty and expense—most plants are home to commensal bacteria that are able to grow rapidly in inexpensive media [80] and are far simpler to engineer than their eukaryotic hosts. Additionally, much like other exogenous strategies bmRNAi is unable to be silenced via RNA-directed DNA methylation. Previous systems in insects have also shown that the silencing produced by dsRNA-expressing symbiotes is both long lasting and systemic, and in select cases, horizontally transmissible [43].

Bacterial-mediation, as an exogenous delivery technology is also partially resistant to degradation by the host prior to inducing an effect. This trait makes bmRNAi highly suitable as a replacement for HIGS—in which pests or pathogens are deterred, inhibited, or killed by dsRNA present in the host organism. HIGS, while technically possible via genome-based expression, is most efficient when dsRNA is produced in plastids—which do not express genes associated with RNA-PTGS [41]. Similarly, delivery bacteria, once depleted of dispensable double-stranded RNAses—namely, RNAse III—are also able to accumulate and compartmentalize silencing RNA [12]. This prevents the processing of the dsRNA by plant pathways prior to entry into the desired organism, thereby allowing for more efficient assembly of RISCs by the target [81,82].

While it is presently unclear how bacteria transmit silencing RNA, it is known that they, much like eukaryotes, can secrete various types of non-coding RNA (ncRNA), collectively referred to as extracellular RNA (exRNA), into the extracellular milieu by producing outer membrane vesicles (OMVs). These vesicles appear to carry a differential selection of cytoplasmic contents and are enriched in ncRNA—such as rRNA and tRNA relative to the cytosol. In addition, there is also evidence that bacteria secrete and release RNA through numerous other means [83].

The exact function of exRNA is still poorly understood, although some hints as to their role can be seen in previous studies of the activity of OMVs. These function through endocytosis, or the fusion of the nascent vesicle with a recipient membrane, at which point the contents are released into the cytoplasm [84]. OMVs have been shown to mediate a variety of interactions between bacteria and neighboring cells—some of which are of profound medical and agricultural significance—although the role of ncRNA in these is not yet clear [85,86].

OMVs may also be of relevance to bmRNAi, and merit further investigation—one could surmise from both size and functional similarity to RNA secretion systems in eukaryotes—which have been shown to harbor micro RNA [87], that they could contain dsRNA along with other ncRNA. Hypothetically, OMVs would not only assist in partitioning dsRNA away from extracellular ribonucleases increasing its persistence in the plant, but also facilitate its uptake into eukaryotic cells [84]. Moreover, such a mechanism would provide some explanation for the greater efficacy of bacterially-delivered dsRNA relative to unpackaged or purified exogenous dsRNA [43].

The development process for bmRNAi typically involves selecting and modifying an appropriate delivery bacterium and then introducing an expression construct, such as a plasmid, to allow the bacteria to produce silencing RNA in association with a host. The choice of delivery bacterium can be critical depending on the design of the system. Long term applications—such as with symbiote-mediation, require the bacterium to replicate in vivo—this requires an understanding of the recipient microbiome, and potential interactions with the host to achieve the best results [43]. This also provides a powerful means of improving and controlling a silencing effect—specialized assemblages, such as endophytic bacteria, can penetrate host tissues more extensively than those lacking the associated adaptations [88,89]. In contrast to this, short-term, or single applications—such as where the bacterium dies after being internalized, are less sensitive to this consideration, and can instead utilize generalized, or non-specialized bacteria [90].

Additionally, the use of plasmid-based dsRNA expression permits modular modification of the system. One can re-target the bacterial strain with simple subcloning and bacterial transformation, allowing for multiple targets or dsRNA designs to be tested simultaneously. Likewise, new promoter configurations, or expression-system specific promoters, such as the T7 polymerase promoter and its cognate polymerase, are equally easy to include in plasmid backbones. Beyond this, plasmids can also be used to encode genes to improve their stability in absence of selection—such as with plasmid partitioning [91] or facilitate RNA uptake—by the addition of symbiotic or pathogenic determinant genes [90].

There have been a few subtle variations in how bacterium-mediated silencing has been previously achieved. Past researchers have worked from two directions; the most common involves having bacteria synthesize the dsRNA from a plasmid or an inserted gene. The bacterium is then applied to the host, where it secretes, or deposits dsRNA to be taken up via endocytosis, membrane transport or secretion into the cytosol subsequently delivering the RNA payload. This method is enhanced by the deletion of *rnc*, the gene responsible for bacterial RNase III, failure to do so results in dramatic reductions in the dsRNA yield, due to degradation into non-active forms [78,79,92,93].

Delivery bacteria can be modified or selected to meet the requirements of specific applications such as controlling when and where the bacteria, or expression plasmid can replicate and, more importantly, how far they are able to penetrate the host. A previously published bmRNAi system, trans-kingdom RNAi (tkRNAi), exemplifies this capacity by using a plasmid to provide non-pathogenic, auxotrophic *E. coli* with the genes required to enter and deliver sRNA [90]. 

In the prototype tkRNAi system, auxotrophic *E. coli* was transformed with plasmids that encode a short hpRNA (shRNA)-generating gene, as well as genes for two proteins to assist with internalization—Invasin (initially derived from *Yersinia pseudotuberculosis)* and Listeriolysin O (initially from *Listeria monocytogenes)*. Once applied to the host, the presence of Invasin allowed the bacteria to be internalised via endocytosis, at which point they were released into the cytoplasm through the action of Listeriolysin O—which creates pores in the endosomal membrane. Once inside, bacteria failed to thrive due to engineered auxotrophy and released their shRNA payload. In this way, researchers were able to induce systemic downregulation of genes in xenografted human intestinal tumors—through both oral and intravenous routes, with minimal side effects [90]. 

In contrast to tkRNAi, another class of previous bacterium-mediated RNAi system instead exploited the capacity of naturally-invasive, tumor-suppressing bacteria to insert DNA capable of transient expression. Test systems for this concept have utilized disarmed, auxotrophic *Salmonella* spp. transformed with a shuttle vector carrying an sRNA gene under a eukaryotic promoter. Once prepared, the bacterial strain is applied to the host, where natural pathogenesis pathways allow them to enter and transfer the vector to the host [94,95,96,97,98].

When internalized, the inserted vector allows the host to transiently express dsRNA, resulting in endogenous production of siRNAs. In this fashion, it was possible to temporarily produce dsRNA in the host using a simple exogenous application. The most current demonstration of this technique, reviewed successfully, demonstrated siRNA-mediated apoptosis of the xenografted prostate cells—although the current state of research into this technique is unknown to the author [93,98].

A recent, striking demonstration involving insects termed symbiote-mediated RNAi improves on previous feeding-induced and bacterium-mediated strategies by using RNAse III-deficient intestinal symbiotes to express and accumulate dsRNA. The modified bacteria are applied to the insects via feeding and colonize the midgut, where they can continually synthesize and release dsRNA that targets genes within the insect. The dsRNA is then absorbed by the insect either through transmembrane channels or endocytosis and subsequently spread through the haemocoel, producing a systemic silencing effect. These bacteria are also highly persistent—it was shown that populations were able to remain in the host for up to 250 days after ingestion. An additional benefit of this method due to the feeding habits of the insects in the test system, is that the bacteria are horizontally transmissible, that is, capable of colonizing uninfected insects via the inadvertent ingestion of infected feces [43].

The test system for this concept was highly successful, the authors of the study experimented on two insect pest species—*Rhodnius prolixus*, a trypanosome-transmitting assassin bug, and *Frankliniella occidentalis*, the western flower thrip, an invasive agricultural pest. In both cases they were able to produce sustained systemic silencing. *R. prolixus* was delivered dsRNA targeting Vg, encoding Vitellogenin, a protein critical to oogenesis, resulting in 72% reduction in the ecclosion rates of eggs laid by infected individuals. In *F. occidentalis* they instead targeted α-tubulin which produced a significant mortality phenotype in both larval and adult individuals—an effect not seen when performed with heat-killed bacteria [43].

These results have broad implications for pest control in terms of both agriculture and public health and are an indication that bmRNAi is maturing to the point of becoming a useful therapeutic. Similar systems could hypothetically be created for other insect species, targets and contexts to address a range of issues and present a timely replacement for older, broad-spectrum treatments if applied correctly. 

## 4. BmRNAi in Plant Protection

As discussed in previous sections, RNAi has tremendous potential as a means for engineering novel crop protection. RNAi is specific, and the mechanisms central to it are present in all higher organisms to some extent. As such, it is reasonable to believe that species-specific biocontrol agents could someday be created for virtually any situation. Despite this, the application of RNAi to this end has been restricted to the transformation of plants, which, in addition to requiring more resources, lacks the ability to dynamically respond to threats due to the time required to produce transgenic plants. As shown in previous sections, there have been many preliminary successes using exogenous RNAi which have highlighted the potential for externally applied RNA biocides. While these gains have been highly promising, these have been limited in their application as real-world crop protectants, and the potential for RNAi is yet to be fully realized in this context.

Bacterially mediated RNAi could provide an intermediate option to previous delivery methods by allowing for a semi-permanent silencing effect to be induced by exogenous application. An RNAi plant symbiote may also add both greater penetration and durability to RNA-based treatments by colonizing the host plant and synthesizing protective dsRNA in situ, in addition to providing a durable and non-toxic exogenous vessel for dsRNA on external surfaces. Plant-associated endophytic bacteria—or those that can replicate inside plant tissues and cells—have significant potential in this respect. These can occupy deep inside plant tissue, including inside cells, and in some cases, adhered to the nuclear membrane [89]. Moreover, many species are capable of systemic colonization from external surfaces, allowing for spray or soaking based administration to be used [99].

Many bacterial symbiotes are also capable of bestowing additional benefits to their hosts, including resistance to pests and pathogens through the production of antibiotic, antimycotic, and herbivore deterring proteins and metabolites [100,101,102]. These could be stacked or reinforced with the addition of an appropriate RNAi strategy to increase the burden to the pest or reduce resistance [40]. In this manner, bacterial-mediation could allow the realization of RNAi as a biocide, by making application cheap and flexible, while facilitating the development of more powerful combined synergistic control agents. These benefits aside, there are undoubtedly still many situations that will be better suited to the use of either HIGS or ExoRNAi, instead of bmRNAi, such as when permanent or short-term, reversible silencing is required, some of these potential advantages and limitations are summarized in Table 2.

Experiments by the authors (Goodfellow et al., unpublished) have shown that engineered strains of a common endophytic laboratory bacterium can induce the medium-term, systemic, and significant silencing of a plant-expressed reporter gene (GUS). Bacteria were infiltrated into a single mature *Nicotiana tabacum* leaf per plant, which was accompanied by systemic silencing that increased in strength over the course of the 21-day experiment. Moreover, it was shown that endophytic bacteria were more efficient at delivery than *E. coli*, producing an equal silencing effect with a much lower dsRNA yield. 

These results underline the potential for symbiotic bmRNAi in plants and lead to the development of a hypothetical model for the bacterial delivery of dsRNA, although many aspects, while supported by the literature, require specific experimental verification. The first logical steps—colonization of the plant, as well as production and reduced degradation of dsRNA by an RNAse III-deficient delivery bacterium—were demonstrated in the experiment. Beyond this, previous research has elucidated numerous steps separately and allowed for some assumptions to be made about the potential dynamics of a symbiotic bmRNAi system in plants.

It has been shown that many bacteria, including the one used in the experiment, are capable of endophytic colonization of *N. tabacum* and can grow into tissue distal from the site of initial infection by colonizing the plants vascular system—specifically, the xylem [99]. During this association, the RNAi bacteria release or secrete hpRNA into both the extracellular milieu, as well as directly into plant cells [86,87]. Once internalized, both the bacteria and the effector RNA can move between adjacent cells via plasmodesmata and eventually into vascular circulation [30,31,103]. Once taken into plant cells, dsRNA is cleaved by Dicer-like proteins to generate primary siRNA that can also induce secondary siRNA synthesis by RdRPs. Both primary and secondary siRNA, once produced, are packaged into RNA-induced silencing complexes and enact the desired effect—sequence-specific gene silencing [5]. 

**Figure 1:**
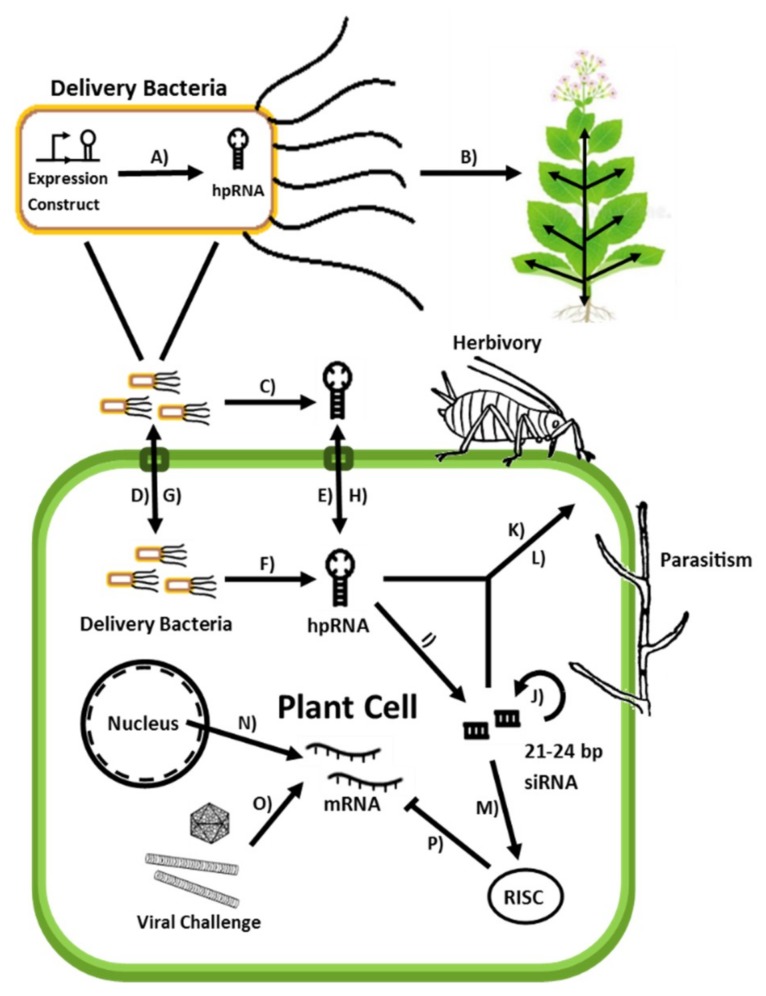
Schematic of processes and elements pertinent to bmRNAi crop protection A) hpRNA is transcribed from an expression construct within an endophytic bacterium. B) Bacteria applied by both invasive and non-traumatic administration systemically colonize the plant host via the vascular system. C) Bacteria secrete hpRNA into the extracellular milieu via lysis or secretion. D) Delivery bacteria present in the hosts’ extracellular matrix are internalized and colonize the cytoplasm. E) hpRNA present in the external environment are internalized via endocytosis. F) Bacteria secrete hpRNA into the cytoplasm via lysis or secretion. G) Bacteria are transferred between adjacent cells and into systemic circulation via xylem plates. H) hpRNA and siRNA produced locally are spread systemically via vascular transport I) Dicer-like proteins cleave hpRNA into 21–24 bp siRNA. J) Primary siRNA triggers secondary siRNA production via RNA-dependent RNA polymerases. K) hpRNA, siRNA and delivery bacteria are ingested by invertebrates feeding on treated plant tissue. L) hpRNA and siRNA are absorbed by fungi parasitizing the plant host. M) siRNA is loaded into RNA-induced silencing complexes (RISCs). N) Plant mRNA is transcribed and released into the cytosol. O) Viral mRNA and dsRNA are produced by viral replication and transcription. P) Cytosolic RISCs degrade and sequester complementary RNA, preventing translation and thereby silencing plant-expressed genes.

With respect to crop protection, the RNA synthesized by both the bacteria and secondarily by the plant host would hypothetically be able to exert a protective effect. As detailed, how this might occur differs with respect to the threat, viruses are fought using the plants endogenous PTGS machinery, whereas invertebrates and fungi instead need to ingest the bacteria or dsRNA to generate an effect. Once ingested, the dsRNA delivery bacteria would presumably be digested by the attacking organism, releasing their dsRNA payload along with that present in the host plant, silencing the target gene. A broad schematic of these processes with respect to crop protection is detailed in Figure 1.

## 5. Conclusions and Further Perspectives

As demonstrated, RNAi has significant potential as a platform for the development of customizable and highly specific biocontrol agents, a goal which has, to date, failed to be fully realized, due to the constraints of available application methods. Exogenous and endogenous delivery strategies, while now substantially developed and of great utility to researchers, still suffer from their respective limitations. Transgenic dsRNA-expressing plants are slow to produce, in addition to being subject to self-silencing by methylation and the premature degradation of effectors by endogenous gene silencing pathways. In contrast, exogenous strategies, while more flexible are also restricted—both RNA degradation and uptake limit the accumulation of meaningful levels of protective RNA in the plant and require regular re-application.

Bacterium-mediated RNAi, while only recently considered in this respect, can cautiously be viewed as a potential improvement by simultaneously increasing the duration and strength of silencing produced by exogenous applications. Once demonstrated for RNAi, GMO delivery bacteria could also be used to express pest/pathogen resistance proteins/chemicals, improving the protection afforded. One key aspect in the development of future systems will be engineering optimally expressing and delivering bacteria. This will require greater understanding of bacterial RNA processing and secretion, as well as the effect that plant-microbial interactions may have on their delivery. 

Another potential issue with bacterial delivery are the environmental risks associated with the release of GMO bacterium—however strategies already exist to prevent the spread of GMO bacteria which should allow for controlled application of these technologies [104,105]. Beyond this, plant-associated RNAi bacteria remain to be specifically tested for the purposes of crop protection, which may differ substantially from the silencing of transgenes or endogenous genes in plant hosts. Very little is also known about the potential for the transmission of RNAi effectors from endophytes to plant parasites, although the research that does exist suggests that we should be optimistic. Nonetheless, the development of this method could be of substantial benefit to both researchers and as a means of protecting plants, although a great many questions and challenges remain to be resolved.

## Figures and Tables

**Table 1:** Experimental gene silencing targets for RNAi-based crop protection.

	Target ^1^	Host Plant	Gene ^2^	Function	Effect	Reference
**Insects**	BMSB	*Phaseolus vulgaris*	Vg	Egg Yolk Precursor	Reduced fertility	[28]
	CB	*Gossypium sp.*	JHAMT	Juvenile Hormone Biosynthesis	Reduced survival and growth	[40]
	CPB	*Nicotiana tabacum*	*Actin*	Cytoskeleton Component	Reduced growth, lethal to larvae	[41]
	WCR	*Zea mays*	V-ATPase	Membrane Transport	Reduced growth, lethal to larvae	[42]
	WFT	Direct symbiote mediation	*Tub*	Cytoskeleton Component	Reduced survival	[43]
**Virus**	PMMoV	*Nicotiana benthamiana*	ORF2	Replicase	Antiviral immunity	[8]
	PVY	*Solanum tuberosum*	HCPro	Protease	Antiviral immunity	[44]
	TMV	*Nicotiana tabacum*	CP	Coat Protein	Antiviral immunity	[45]
	BCMV	*Nicotiana benthamiana*	Nib	Nuclear Inclusion	Antiviral immunity	[9]
**Fungi**	Fg	*Hordeum vulgare*	CYP51	Membrane biosynthesis	Reduced fungal growth	[10]
	Bc	*Solanum lycopersicum*	DCL1/DCL2	Host Immunity suppression	Reduced pathogenicity	[46]
	Foc	*Musa sp. cv. Rasthali*	FTF1	Pathogenesis	Reduced pathogenicity	[47]
	Vd	*Gossypium sp.*	VdH1	Microsclerotia formation	Reduced pathogenicity	[48]

^1^ BSMB—Brown Marmorated Stink Bug, CB—Cotton Bollworm, CPB—Colorado Potato Beetle, WCR—Western Corn Rootworm, WFT—Western Flower Thrip, PMMoV—Pepper Mild Mottle Virus, PVY—Potato Virus Y, TMV—Tobacco Mosaic Virus, BCMV—Bean Common Mosaic Virus, Fg—*Fusarium graminearum*, Bc—*Botrytis cinerea*, Foc—*Fusarium oxysporum f. sp. Cubense*, Vd—*Verticillium dahliae*. ^2^ Vg—Vitellogenin, JHAMT—juvenile hormone acid O-methyltransferase, Actin—Actin, V-ATPase—Vacuolar ATPase, Tub—β-tubulin, ORF2—PMMoV Replication-associated protein, HCPro—PVY Protease, CP—TMV Coat Protein, Nib—Nuclear inclusion protein b, CYP51—cytochrome P450 lanosterol C-14 α-demethylase, DCL1/DCL2—Dicer like protein 1/2, FTF1—Fusarium Transcription Factor 1, VdH1—Hygrophobins 1.

**Table 2:** Advantages and limitations of RNAi delivery methods for silencing-based crop protection.

Application Method	Advantages/Potential Advantages	Limitations
Endogenous/Host-Induced RNAi	Permanent Inheritable Extensively tested	RNAi plants are subject to GMO regulation Transformation not possible in all plant species Costly and time consuming to produce Low level accumulation of long dsRNA without plastid transformation Self-silencing attenuates expression of dsRNA over time
Exogenous RNAi	Applicable without host transformation Numerous administration methods RNA backbone modifications possible Diverse synthesis methods Easily customised Potentially non-GMO	Requires frequent reapplication for persistent silencing Limited by uptake and degradation of RNA Large scale production of RNA expensive
Bacterium-mediated RNAi	Semi-permanent Stronger systemic silencing Improved dsRNA penetration and uptake RNA protected from degradation Can be retargeted easily No self-silencing	May require reapplication (at a lower frequency than Exo-RNAi) Requires mechanisms to prevent escape of GMO bacteria Symbiote-mediated RNA-delivery poorly understood in plants Requires modification of suitable endophytic bacteria
